# Effects of changes in veno-venous extracorporeal membrane oxygenation blood flow on the measurement of intrathoracic blood volume and extravascular lung water index: a prospective interventional study

**DOI:** 10.1007/s10877-022-00931-0

**Published:** 2022-10-25

**Authors:** Alice Marguerite Conrad, Gregor Loosen, Christoph Boesing, Manfred Thiel, Thomas Luecke, Patricia R. M. Rocco, Paolo Pelosi, Joerg Krebs

**Affiliations:** 1 Department of Anaesthesiology and Critical Care Medicine, University Medical Centre Mannheim, Medical Faculty Mannheim of the University of Heidelberg, Theodor-Kutzer Ufer 1-3, 68165 Mannheim, Germany; 2 Department of Cardiothoracic Anaesthesia and Intensive Care, Royal Papworth Hospital NHS Foundation Trust, Papworth Road, Cambridge Biomedical Campus, Cambridge, CB2 0AY UK; 3 Laboratory of Pulmonary Investigation, Carlos Chagas Filho Institute of Biophysics, Federal University of Rio de Janeiro, Centro de Ciências da Saúde, Avenida Carlos Chagas Filho, 373, Bloco G-014, Ilha Do Fundão, Rio de Janeiro, Brazil; 4 Department of Surgical Sciences and Integrated Diagnostics, University of Genoa, Genoa, Italy; 5 Anesthesia and Intensive Care, San Martino Policlinico Hospital, IRCCS for Oncology and Neurosciences, Genoa, Italy

**Keywords:** Acute respiratory distress syndrome, Extracorporeal membrane oxygenation, Transpulmonary thermodilution, Intrathoracic blood volume index, Extravascular lung water index

## Abstract

In severe acute respiratory distress syndrome (ARDS), veno-venous extracorporeal membrane oxygenation (V-V ECMO) has been proposed as a therapeutic strategy to possibly reduce mortality. Transpulmonary thermodilution (TPTD) enables monitoring of the extravascular lung water index (EVLWI) and cardiac preload parameters such as intrathoracic blood volume index (ITBVI) in patients with ARDS, but it is not generally recommended during V-V ECMO. We hypothesized that the amount of extracorporeal blood flow (ECBF) influences the calculation of EVLWI and ITBVI due to recirculation of indicator, which affects the measurement of the mean transit time (MTt), the time between injection and passing of half the indicator, as well as downslope time (DSt), the exponential washout of the indicator. EVLWI and ITBVI were measured in 20 patients with severe ARDS managed with V-V ECMO at ECBF rates from 6 to 4 and 2 l/min with TPTD. MTt and DSt significantly decreased when ECBF was reduced, resulting in a decreased EVLWI (26.1 [22.8–33.8] ml/kg at 6 l/min ECBF vs 22.4 [15.3–31.6] ml/kg at 4 l/min ECBF, p < 0.001; and 13.2 [11.8–18.8] ml/kg at 2 l/min ECBF, p < 0.001) and increased ITBVI (840 [753–1062] ml/m^2^ at 6 l/min ECBF vs 886 [658–979] ml/m^2^ at 4 l/min ECBF, p < 0.001; and 955 [817–1140] ml/m^2^ at 2 l/min ECBF, p < 0.001). In patients with severe ARDS managed with V-V ECMO, increasing ECBF alters the thermodilution curve, resulting in unreliable measurements of EVLWI and ITBVI. German Clinical Trials Register (DRKS00021050). Registered 14/08/2018. https://www.drks.de/drks_web/navigate.do?navigationId=trial.HTML&TRIAL_ID=DRKS00021050

**Supplementary Information:**

The online version contains supplementary material available at 10.1007/s10877-022-00931-0.

## Purpose

Acute respiratory distress syndrome (ARDS) is a clinically challenging condition of pulmonary dysfunction with various cause [1] and it is characterized by formation of edema in alveolar-capillary membrane as well as hypoxemia due to increased transpulmonary shunt [2]. For the most severe cases, veno-venous extracorporeal membrane oxygenation (V-V ECMO) has been proposed as an alternative therapeutic strategy that might reduce mortality [3–5]. A restrictive fluid management is recommended in patients with ARDS managed with V-V ECMO [6, 7] because an excessive positive fluid balance increases mortality [8]. Transpulmonary thermodilution (TPTD) allows us to quantify pulmonary edema from the measurement of the extravascular lung water index (EVLWI) and for continuous monitoring of cardiac preload parameters such as the intrathoracic blood volume index (ITBVI) [9–11], which is mathematically derived from the global end-diastolic volume index (GEDVI). The thermodilution curve is computed using a single-dye indicator method with a bolus of cold saline and is then compartmentalized by the analysis software into the mean transit time (MTt), i.e., the time between injection and passing of half the indicator, and the downslope time (DSt), i.e., the exponential washout of the indicator [12]. ITBVI and EVLWI are then calculated from MTt and DSt. Traditionally, TPTD has not been recommended in patients with ARDS managed with V-V ECMO because of an imputed extracorporeal blood flow (ECBF)-dependent loss and recirculation of the thermo-indicator into the extracorporeal circuit, thus yielding change in the thermodilution curve [13, 14]. Our group previously demonstrated that the amount of ECBF does not influence the lack of interchangeability of comparative cardiac stroke volume measurements with echocardiography and TPTD and the calculated cardiac output (CO_TPTD_) in patients managed with ECMO [15]. The aim of the study was to elucidate the extent of the measurement deviation caused by modulation of ECBF on ITBVI and EVLWI, which typically change over a longer period of time. To our knowledge, no previous prospective studies have investigated the effects of ECBF in V-V ECMO on the MTt and DSt measured by TPTD and the subsequent calculation of ITBVI and EVLWI. In patients with ARDS managed with V-V ECMO, we hypothesized that the calculation of ITBVI and EVLWI with TPTD is influenced by the amount of ECBF resulting in changes in the measurement of MTt and DSt.

## Methods

The study was approved by the local ethics committee (Medizinische Ethikkommission II, University Medical Centre Mannheim, Medical Faculty Mannheim of the University of Heidelberg, Mannheim, registration number 2018-606N-MA) and registered at the German Clinical Trials Register (DRKS00017237). After obtaining written informed consent from the patients or next of kin, we collected prospective data from 20 patients with severe ARDS managed with V-V ECMO admitted to the Department of Anesthesiology and Critical Care Medicine, University Medical Centre Mannheim, Medical Faculty Mannheim of the University of Heidelberg in Mannheim, Germany. The attending physician initiated V-V ECMO therapy according to the standard operating procedures of the department when patients fulfilled the criteria published in the guidelines of the Extracorporeal Life Support Organization [16]. Our institutional management strategy for patients on V-V ECMO support due to primary respiratory failure is outlined in the Supplementary Information. Exclusion criteria for the study were age < 18 years, pregnancy, inherited cardiac malformations, known severe heart valve dysfunctions, end-stage chronic cardiopulmonary failure, and patients with an expected survival of less than 24 h determined by the attending physician. All patients were managed with a central venous catheter inserted via an internal jugular vein and a thermodilution catheter (5F Pulsiocath, Pulsion Medical Systems, Munich, Germany) inserted via a femoral artery [17].

Correct positioning of the central venous catheter was verified by chest radiography as per the standard operating procedure of our unit. During the measurement, patients were sedated with midazolam (5–15 mg/h) and sufentanil (50–250 µg/h) to achieve a Richmond Agitation-Sedation Score of − 5 [18] and received neuromuscular blocking agents (cisatracurium, 6–20 mg/h) to prevent spontaneous breathing efforts [19]. If the mean arterial pressure was below 65 mmHg despite sufficient intravascular volume, continuous norepinephrine infusion was started. In the case of a cardiac index < 2.0 l/min/m^2^ measured by TPTD despite sufficient cardiac pre- and afterload, dobutamine was established in the therapy. Furthermore, we use a standardized echocardiography protocol in accordance with current recommendations to verify the underlying diagnosis [20]. The body surface area was calculated by the TPTD device (PiCCOplus, Pulsion Medical Systems SE, Munich, Germany) according to the formula of Du Bois [21].

Patients were eligible for the study as soon as an ECBF of 2 l/min generated a partial pressure of oxygen of at least 60 mmHg with standardized respiratory settings (respiratory rate of 12–16/min, tidal volume of 3 ml/kg of predicted body weight, positive end-expiratory pressure according to the ARDS Network recommendations [22] and an inspiration-to-expiration ratio of 1:1) on the ventilator (Engström Carestation, GE Healthcare, Munich, Germany). Utilizing this approach and considering patient safety, we were able to modify the ECBF in a wide clinically relevant range.

### Experimental protocol

The ECBF of the V-V ECMO was increased to 6 l/min. After a 15-min equilibration period and in accordance with the recommendations of the manufacturer TPTD measurements of cardiac stroke volume (SV), CO_TPTD_, ITBVI, and EVLWI were then performed 3 times with 20 ml of cold saline (4 °C) (Supplementary Fig. 1). Consecutively, ECBF was reduced to 4 and 2 l/min and a TPTD measurement was repeated after an equilibration period of 15 min. In addition, at each distinct ECBF, respiratory and hemodynamic parameters were recorded. We also measured the arterial pH (pHa), arterial partial pressure of oxygen (PaO_2_), and arterial partial pressure of carbon dioxide (PaCO_2_) with blood gas analysis. Gas flow on the extracorporeal membrane was altered if necessary to maintain a pHa between 7.35 and 7.45.

### Calculations

CO_TPTD_ was derived from TPTD using the Stewart-Hamilton equation [23] and SV subsequently calculated by dividing CO_TPTD_ by the heart rate (HR). Because there is no simple method to acquire MTt and DSt directly at the bedside, we multiplied ITBVI with the body surface area and EVLWI with predicted bodyweight to calculate ITBV and EVLW. Intrathoracic thermovolume (ITTV) was then calculated as EVLW plus ITBV. The global end-diastolic volume (GEDV) was computed as ITBV divided by 1.25, and the pulmonary thermovolume (PTV) was calculated as ITTV minus GEDV. MTt was then calculated as ITTV divided by CO_TPTD_ and DSt as PTV divided by CO_TPTD_ [9, 24].

### Statistical analysis

The number of patients was calculated from preliminary data and it was in line with a previously published study conducted by our group [15]. We assumed a partial η^2^ of 0.11 and an effect size of 0.35. According to a power analysis based on these data, we expected that a sample size of 20 would provide the appropriate power (1 − β = 0.9) to identify significant (α = 0.05) differences. Data describing the influence of ECBF on ITBVI and EVLWI were analyzed with repeated measurement ANOVA followed by Holm-Sidak’s post-hoc test or the Friedman procedure as appropriate and are shown as means ± standard deviation or as medians and interquartile range (25% to 75% interquartile range). We used a mixed effects model with a fixed effect for flow and a subject-specific random effect followed by a post-test for linear trend to analyze the influence of ECBF on ITBVI and EVLWI measurements and MTt and DSt computations. This model uses a compound symmetry covariance matrix and is fit using restricted maximum likelihood. Computation of intra-examination analysis of TPTD measurements has been described previously [25]. Briefly, the coefficient of variation is calculated as the standard deviation of 3 consecutive TPTD measurements divided by their mean. Then, by dividing by the total number of measurements, the coefficient of error is derived. Precision is defined as 2 times the coefficient of error. The least significant change between 2 measurements has been defined as the coefficient of error × 1.96 × √2.

Statistical analyses were performed using Prism Version 8.0.2 (GraphPad Software, San Diego, CA, USA). The level of significance was set at p < 0.05.

## Results

**Fig. 1:**
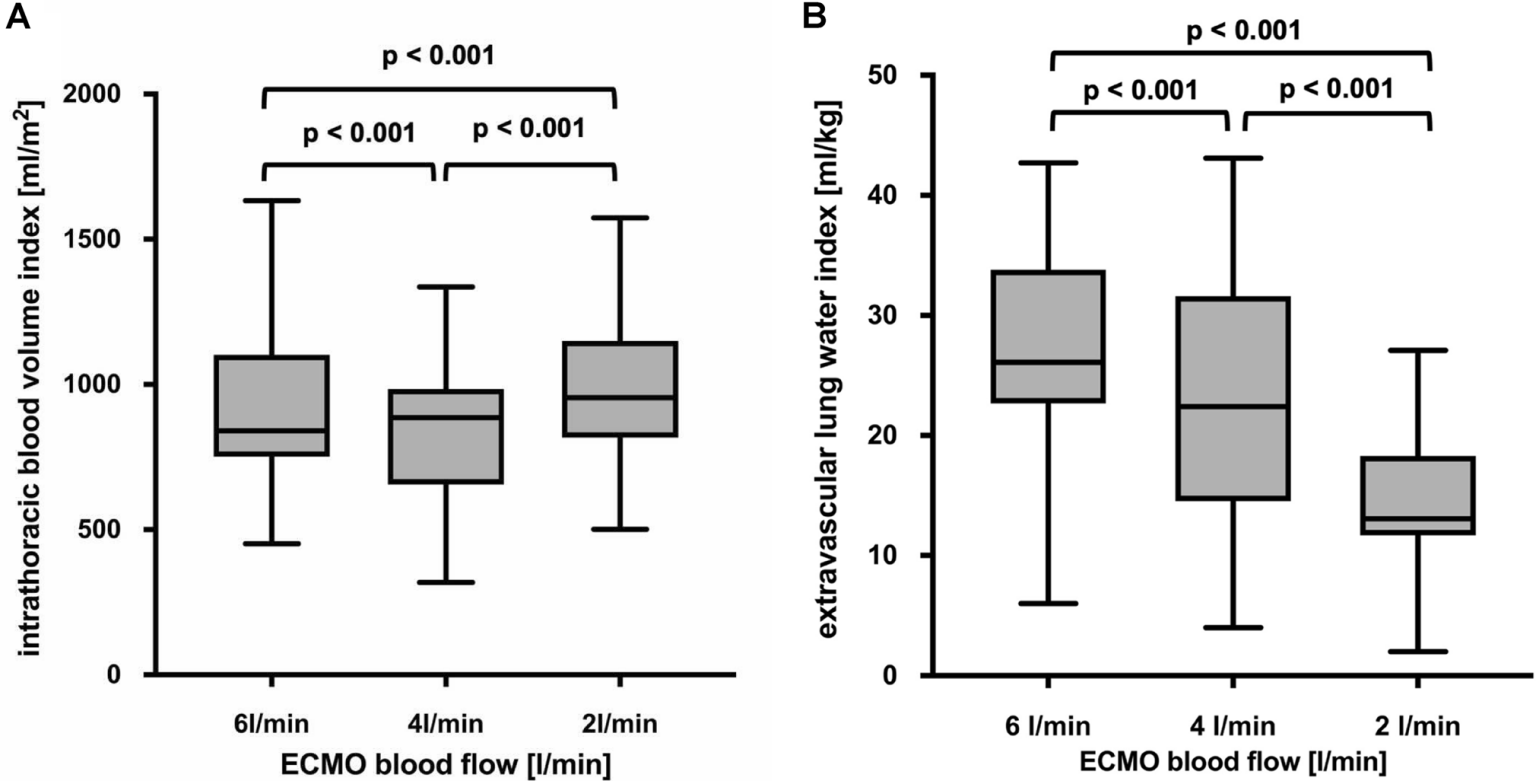
Effects of 3 different extracorporeal blood flows on the intrathoracic blood volume index (**A**) and the extravascular lung water index (**B**) measured with transpulmonary thermodilution. Boxes show the interquartile range (25 to 75%), whiskers encompass the range (minimum–maximum), and horizontal lines represent the median of 20 patients with severe ARDS managed with V-V ECMO. Brackets denote statistically significant differences between different ECBF. p values are shown above the brackets

Twenty patients with severe ARDS managed with V-V ECMO were included in the analysis. The demographic and clinical characteristics of the patients are presented in Table 1. Physiological data at an ECBF of 6, 4 and 2 l/min are provided in Supplementary Table 1. Gradually reducing ECBF from 6 to 2 l/min progressively increased CO_TPTD_ due to an increase in SV and HR. The reduction of ECBF also increased PaCO_2_ with a corresponding decrease in pHa and PaO_2_. Computed ITBVI increased (840 [753–1062] ml/m^2^ at 6 l/min ECBF vs 886 [658–979] ml/m^2^ at 4 l/min ECBF, p < 0.001; and 955 [817–1140] ml/m^2^ at 2 l/min ECBF, p < 0.001, respectively), and EVLWI decreased (26.1 [22.8–33.8] ml/kg at 6 l/min ECBF vs 22.4 [15.3–31.6] ml/kg at 4 l/min ECBF, p < 0.001; and 13.2 [11.8–18.8] ml/kg at 2 l/min ECBF, p < 0.001, respectively) when reducing ECBF from 6 to 4 and 2 l/min (Fig. 1A, B).

**Table 1:** Demographic and clinical characteristics for patients with severe ARDS managed with V-V ECMO

	All patients
Sex [male/female]	7/13
Age [years]	56 ± 13
Height [cm]	173 ± 10
Body weight [kg]	95 ± 35
Body mass index [kg/m^2^]	30 ± 7
Body surface area [m^2^]	2.1 ± 0.4
SAPS II	72 ± 13
SOFA	12 ± 3
RESP score	0 ± 3
ICU Mortality [patients]	4
Mechanical ventilation before study [days]	14 ± 8
Mechanical ventilation before ECMO [days]	4 ± 5
ECMO before study [days]	10 ± 6
Duration ECMO support [days]	15 ± 9
Length of ICU stay [days]	27 ± 12
ARDSp vs. ARDSexp	16/4
Cause of ARDSp	
*Influenza virus*	5/20
*Legionella pneumophila*	2/20
*Streptococcus pneumoniae*	2/20
*Pseudomonas aeroginosa*	1/20
*Varicella zoster*	1/20
*Citrobacter spp.*	2/20
*Staphylococcus aureus*	1/20
No detectable pathogen	2/20
Cause of ARDSexp	
Anastomotic leak with septic shock	2/20
Pancreatitis with septic shock	2/20

*SAPS II* Simplified Acute Physiology Score II, *SOFA* Sequential Organ Failure Assessment; *RESP* Respiratory ECMO Survival Prediction, *PRESERVE* Predicting Death for Severe ARDS on vv-ECMO, *ICU* intensive care unit, *V-V ECMO* veno-venous extracorporeal membrane oxygenation, *ARDS* acute respiratory distress syndrome, *ARDSp* pulmonary acute respiratory distress syndrome, *ARDSexp* extrapulmonary acute respiratory distress syndrome

Data are reported as absolute number or mean ± standard deviation as appropriate

According to the mixed model, ITBVI increased by 18.4 ml/m^2^ (p = 0.0005; confidence interval [CI], 8.23–28.56), and EVLWI decreased by 3.0 ml/kg (p < 0.0001; CI, 2.63–3.45) when decreasing ECBF by 1 l/min. The corresponding calculations for ITBV and EVLW are shown in Supplementary Table 2.

**Fig. 2:**
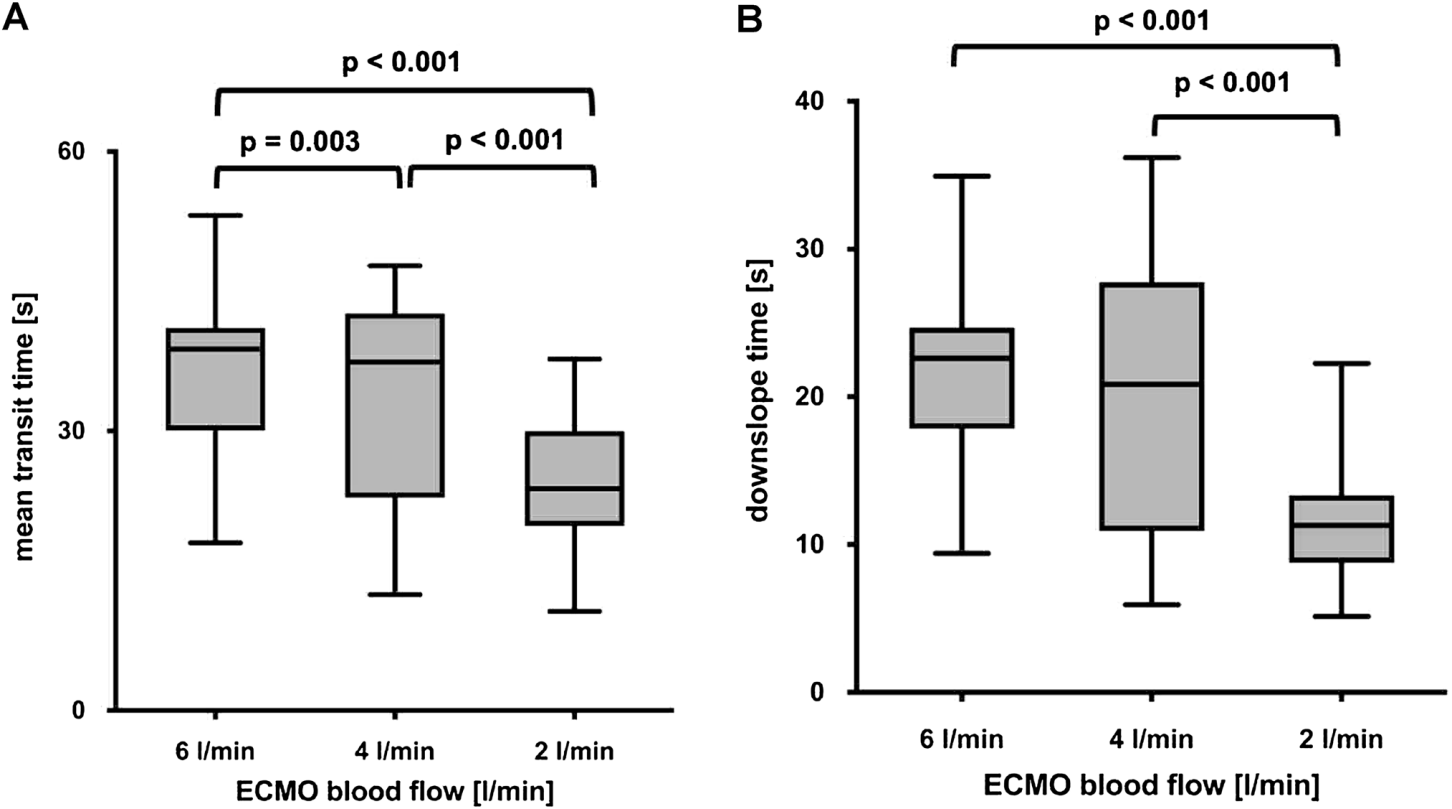
Effects of 3 different extracorporeal blood flows on mean transit time (**A**) and downslope time (**B**) measured with transpulmonary thermodilution. Boxes show the interquartile range (25% to 75%), whiskers encompass the range (minimum–maximum), and horizontal lines represent the median of 20 patients with severe ARDS managed with V-V ECMO. Brackets denote statistically significant differences between different ECBF. p values are shown above the brackets

The calculated values for MTt and DSt decreased significantly when ECBF was reduced from 6 to 4 and to 2 l/min (Fig. 2A, B). The reduction of ECBF by 1 l/min resulted in a decrease of MTt by 2.75 s (p < 0.0001; CI, 2.37–3.13) and DSt by 2.49 s (p < 0.0001; CI, 2.07–2.91).

Computed values for ITTV decreased significantly (3427 [2858–4341] ml at 6 l/min ECBF vs 3309 [2870–3790] ml at 4 l/min ECBF, p < 0.001; and 3035 [2371–3562] ml at 2 l/min ECBF, p < 0.001) and GEDV increased (1325 [1020–1924] ml at 6 l/min ECBF vs 1373 [1039–1725] ml at 4 l/min ECBF, p = 0.009; and 1545 [1196–2043] ml at 2 l/min ECBF, p < 0.001), when reducing ECBF (Table 2). Correspondingly, computed PTV decreased when ECBF was reduced from 6 to 4 l/min (2252 [1994–2240] ml at 6 l/min ECBF vs 1968 [1607–2331] ml at 4 l/min, p < 0.001) and further to 2 l/min (1968 [1607–2331] ml at 4 l/min vs 1378 [1161–1517] ml at 2 l/min, p < 0.001) (Table 2).

**Table 2:** Calculated transpulmonary thermodilution parameters of 20 patients with severe ARDS managed with V-V ECMO at three different extracorporeal blood flows

	ECBF 6 l/min	ECBF 4 l/min	ECBF 2 l/min	ECBF effect
ITTV [ml]	3427 (2858/4341)^**a,b**^	3309 (2870/3790)^**c**^	3035 (2371/3562)	**p < 0.001**
GEDV [ml]	1325 (1020/1924)^**a,b**^	1373 (1039/1725)^**c**^	1549 (1196/2043)	**p < 0.001**
PTV [ml]	2108 (1994/2240)^**a,b**^	1968 (1607/2331)^c^	1378 (1161/1517)	**p < 0.001**

*ECBF* extracorporeal blood flow, *ITTV* intrathoracic thermo-volume, *GEDV* total end-diastolic volume, *PTV* pulmonary thermo-volume

Parameters calculated from transpulmonary thermodilution of 20 patients with severe ARDS managed with V-V ECMO at three different ECBF (25% to 75% interquartile range). The Friedman procedure was used to compare three different vv-ECMO blood flows (*p* < 0.05). Bold numbers represent statistically significant differences between ECBF

**a:** ECBF of **6** l/min vs. ECBF of **4** l/min

**b:** ECBF of **6** l/min vs. ECBF of **2** l/min

**c:** ECBF of **4** l/min vs. ECBF of **2** l/min

The coefficient of variation, coefficient of error, precision, and the least significant change are presented in Supplementary Table 3.

## Discussion

In 20 mechanically ventilated patients with severe ARDS undergoing V-V ECMO support, we evaluated the impact of 3 ECBF rates (6, 4, and 2 l/min) on ITBVI and EVLWI derived by TPTD. We found that (1) the measurements of ITBVI and EVLWI with TPTD are affected by ECBF mainly due to a blood flow-dependent increase in MTt and DSt; (2) ITBVI is less influenced by V-V ECMO blood flow than EVLWI.

To our knowledge, this is the first study prospectively investigating ITBVI and EVLWI under clinical conditions in a selected group of patients with severe ARDS receiving V-V ECMO support. TPTD is easily performed at the bedside, it has a high inter- and intra-observer reliability, and it does not need extensive training and formal education [26, 27]. There are many factors that might affect the thermodilution curve, i.e., changes in body temperature, alterations in the vascular resistance or volume status, volume shift due to inflammatory processes, and changes in the geometry of the heart chambers. Short-term changes in all these factors are unlikely. We tried to control for these confounders in our experimental protocol with a sequential modulation of ECBF and a 15-min equilibration period. To minimize other possible effect of confounders, we used a fixed and predetermined ventilator strategy with low tidal volume according to recent guidelines [28], and conducted the single measurements on one patient sequentially with one type of TPTD device. Therefore, we reduced the influence of possible changes in patient physiology over time and different algorithms in TPTD computers [27].

In case of thermo-indicator loss into the extracorporeal circuit and delayed dispersion into the patient’s circulation, V-V ECMO resembles an intracardiac left-to-right shunt. Significant anatomic intracardiac left-to-right shunt influences TPTD curves [29], largely increasing EVLWI values with more pronounced alteration of the decreasing slope of the thermodilution curve. Giraud et al. reported that intracardiac left-to-right shunt causes thermo-indicator recirculation which increases DSt and to a lesser extent MTt [29]. In line with their report, we found an increase in computed MTt of 2.75 s and a respective increase in computed DSt of 2.49 s per 1 l/min of ECBF with a significant increase in EVLWI. Some distinctive differences between an anatomic shunt and the extracorporeal circuit should be noted: first, recirculation is only present on the right side of the heart without direct impact of rapid changes of filling conditions throughout the cardiac cycle; second, an additional flow and pressure-generating pump is part of the V-V ECMO setup; and third, the circuit itself represents a large additional distribution volume for the thermo-indicator [30]. Because DSt describes the exponential indicator outwash in the patient’s arterial circulation, it is likely affected by alterations caused by V-V ECMO because the thermo-indicator for TPTD might recirculate within the extracorporeal circuit and then be distributed in a prolonged and delayed manner to the arterial thermodetector. CO_TPTD_ measured by TPTD is inversely correlated to the total area under the curve. Thus, a massively prolonged DSt would result in an increased area under the curve, which corresponds to slow distribution of thermo-indicator and therefore a low CO_TPTD_. We observed a similar impact on CO_TPTD_, but not at the same magnitude as indicated by the enlarged area under the curve that would result from an increase in MTt and DSt. This could be due to the mathematic correction performed by the PiCCO software that takes a certain amount of recirculation into account [31, 32]. This finding is in line with our previous report, where we found no influence of ECBF on the difference in measurements of cardiac SV by echocardiography and TPTD in patients with severe ARDS managed with V-V ECMO [15]. Comparative measurements of cardiac SV between TPTD and echocardiography are not interchangeable [15]. Other methods of cardiac SV measurements either use some other form of indicator potentially susceptible to recirculation or are not validated in patients on ECMO [33, 34]. Thus, our data are not appropriate to differentiate between real changes in CO due to ECBF and corresponding delivery of oxygen modulations, or MTt and especially DSt prolongation due to recirculation.

Our results differ from those reported by Haller et al., who found increased CO measured by dye dilution TPTD at a higher ECBF [13]. They compared a conventional CO_TPTD_ measurement method with a dye dilution method but did not investigate ITBVI or EVLWI. The ECBF at the measurements was also not reported [13]. These differences may be at least in part explained by variations in the setup of the extracorporeal circuit: first, they investigated a circuit setup with 2 oxygenators increasing the extracorporeal volume; second, in their investigation, small bore cannulas were used, which typically increase the suction pressure on the drainage cannula promoting recirculation [35]. Decreasing ECBF by 1 l/min increased ITBVI by 18.4 ml/m^2^ and decreased EVLWI by 3.0 ml/kg/m^2^. Due to the respective calculations (outlined in the Supplementary Information), MTt and DSt affect the calculation of ITBVI in equal ratio, whereas DSt contributes 5 times as much to the calculation of EVLWI than MTt. A previous sub-study showed no significant differences between comparative GEDVI and CO measurements at an ECBF of 6, 4, 2, and 0 l/min in 7 patients with severe ARDS, supported by V-V ECMO [15]. However, our current study investigates almost 3 times as many patients and therefore might be better suited to describe changes in GEDVI, ITBVI and CO_TPTD_ due to ECBF modulation. Herner et al. reported that CO_TPTD_ decreased (but not by pulse contour analysis), whereas GEVDI and EVLWI increased after initiation of V-V ECMO with an ECBF of approximately 3 l/min in 14 patients [36]. They hypothesized that GEDVI and EVLWI increased due to an alteration of the measurement of DSt. This is in line with our findings, showing that higher ECBF rates prolong MTt and DSt, resulting in a clinically relevant miscalculation of EVLWI because the overestimation of pulmonary edema might promote inappropriate use of diuretics or dialysis. On the other hand, the underestimation of ITBVI by 18.4 ml/m^2^ per l/min of ECBF should not influence the attending physician to change the clinical management because static preload parameters are generally not recommended to predict fluid responsiveness and fluid demands in critically ill patients [37]. Central venous pressure, another surrogate parameter for static cardiac preload, did not change due to modulation of ECBF. Thus, recirculation of the thermo-indicator in the V-V ECMO circuit might represent a confounder of the measurements, which fundamentally violates assumptions of TPTD [38]. Quantifying recirculation at the bedside is difficult [30] and requires either the measurement of mixed venous saturation using a pulmonary catheter [39] or pausing the membrane gas flow [30, 40, 41]. Measuring the oxygen saturation in front of the oxygenator is not recommended for calculation of recirculation in clinical setting [42]. Increasing ECBF almost always results in an increase in recirculation [43, 44], and thus offers a valid explanation for our results and the change observed in the shape of the thermodilution curve. All measurements were performed in an individual patient consecutively within a short time interval, thus it is unlikely that substantial change in patient physiology, such as the amount of cardiac preload or extravascular lung edema, occurred in the observation period. In patients with severe ARDS managed with V-V ECMO, TPTD measurements of MTt and DSt are affected by the amount of ECBF, resulting in a clinically relevant overestimation of EVLWI. ITBVI also underlies a miscalculation due to higher ECBF, but to a smaller extent and thus is not relevant for clinical practice.

### Limitations

Several limitations of our study need to be acknowledged. First, a direct measurement of the recirculation in the V-V ECMO circuit was not performed, because we did not use a pulmonary catheter or stop the membrane gas flow over a prolonged timespan with regard to patient safety [30, 40]. Therefore, we can only speculate about the underlying mechanisms causing the observed alterations in the thermodilution curve. Second, TPTD measurements were not obtained without ECBF after termination of the V-V ECMO therapy. Therefore, our data do not allow for extrapolations of ITBVI and EVLWI below the reported range of ECBF. However, we tested clinically relevant ECBF that might enable the clinician to estimate the true cardiac preload and the amount of extravascular lung edema. In this regard, our results can generate a hypothesis for a longitudinal clinical study. Third, due to the profound influence of different cannula and circuit designs, we cannot extrapolate our results to any configuration other than a femoral-jugular cannulation strategy. The same applies for the use of different TPTD devices that might use other calculation algorithms. Fourth, we did not compare ITBVI and EVLWI measurements after ECBF modulations with a reference method such as echocardiography or serial computed tomography. Fifth, the number of patients included in the study is limited and this may influence the validity of our results.

## Conclusions

In patients with severe ARDS managed with V-V ECMO, increasing ECBF alters the thermodilution curve, resulting in unreliable measurements of EVLWI and ITBVI. These parameters should be interpreted cautiously in ARDS patients managed with V-V ECMO.

## Supplementary Information

Below is the link to the electronic supplementary material.

Supplementary file1 (DOCX 108 KB)

## Data Availability

The datasets used and/or analyzed during this study are available from the corresponding author on reasonable request.

## Abbreviations

ARDS: Acute respiratory distress syndrome
CO_TPTD_: Cardiac output measured by transpulmonary thermodilution
CI: Confidence interval
CVP: Central venous pressure
DSt: Downslope time
ECBF: Extracorporeal blood flow
EVLW: Extravascular lung water
EVLWI: Extravascular lung water index
GEDV: Global end-diastolic volume
HR: Heart rate
ITBV: Intrathoracic blood volume
ITBVI: Intrathoracic blood volume index
ITTV: Intrathoracic thermovolume
MTt: Mean transit time
pHa: Arterial pH
PaCO_2_: Arterial partial pressure of carbon dioxide
PaO_2_: Arterial partial pressure of oxygen
PTV: Pulmonary thermovolume
SV: Stroke volume
TPTD: Transpulmonary thermodilution
V-V ECMO: Veno-venous extracorporeal membrane oxygenation
