# Supplementary material for: Effects of changes in veno-venous extracorporeal membrane oxygenation blood flow on the measurement of intrathoracic blood volume and extravascular lung water index: a prospective interventional study
Source: J Clin Monit Comput. 2022 Oct 25;37(2):599–607. doi: 10.1007/s10877-022-00931-0 (PMC9595580; doi:10.1007/s10877-022-00931-0)
Supplement: Supplementary file 1 — Supplementary file1 (DOCX 108 KB) [file 10877_2022_931_MOESM1_ESM.docx]

Effects of changes in veno-venous extracorporeal membrane oxygenation blood flow on the measurement of intrathoracic blood volume and extravascular lung water index: a prospective interventional study

Alice Marguerite Conrad^1^*, Gregor Loosen^2^*, Christoph Boesing^1^, Manfred Thiel^1^, Thomas Luecke^1^, Patricia R.M. Rocco^3^, Paolo Pelosi^4, 5^, Joerg Krebs^1^

^1^Department of Anaesthesiology and Critical Care Medicine, University Medical Centre Mannheim, Medical Faculty Mannheim of the University of Heidelberg, Mannheim, Germany, Theodor-Kutzer Ufer 1-3, 68165 Mannheim, Germany

^2^Department of Cardiothoracic Anaesthesia and Intensive Care, Royal Papworth Hospital NHS Foundation Trust, Cambridge, United Kingdom, Papworth Road, Cambridge Biomedical Campus, CB2 0AY, Cambridge, UK

^3^Laboratory of Pulmonary Investigation, Carlos Chagas Filho Institute of Biophysics, Federal University of Rio de Janeiro, Centro de Ciências da Saúde, Avenida Carlos Chagas Filho, 373, Bloco G-014, Ilha do Fundão, Rio de Janeiro, Brazil

^4^Department of Surgical Sciences and Integrated Diagnostics, University of Genoa, Genoa, Italy

^5^Anesthesia and Intensive Care, San Martino Policlinico Hospital, IRCCS for Oncology and Neurosciences, Genoa, Italy

*****Alice Marguerite Conrad and Gregor Loosen equally contributed to this work

Corresponding author: Joerg Krebs, Department of Anaesthesiology and Critical Care Medicine, University Medical Centre Mannheim, Theodor-Kutzer-Ufer 1-3, 68167 Mannheim, Germany. E-mail: [joerg.krebs@umm.de](mailto:Joerg.Krebs@umm.de). ORCID: 000-003-3037-0144

E-mail addresses:

Alice Marguerite Conrad: alice.conrad@umm.de

Gregor Loosen: gregor.loosen@nhs.net ORCID: 0000-0001-7441-9726

Christoph Boesing: christoph.boesing@umm.de

Manfred Thiel: manfred.thiel@umm.de

Thomas Luecke: thomas.luecke@medma.uni-heidelberg.de

Patricia RM Rocco: [prmrocco@biof.ufrj.br](mailto:prmrocco@biof.ufrj.br) ORCID: 000-003-1412-7136

Paolo Pelosi: [ppelosi@hotmail.com](mailto:ppelosi@hotmail.com) ORCID: 0000-0001-5055-3023

**SI figure 1:** Flowchart of the experimental protocol. Measurement 20 patients with severe ARDS managed with V-V ECMO at three different extracorporeal blood flows


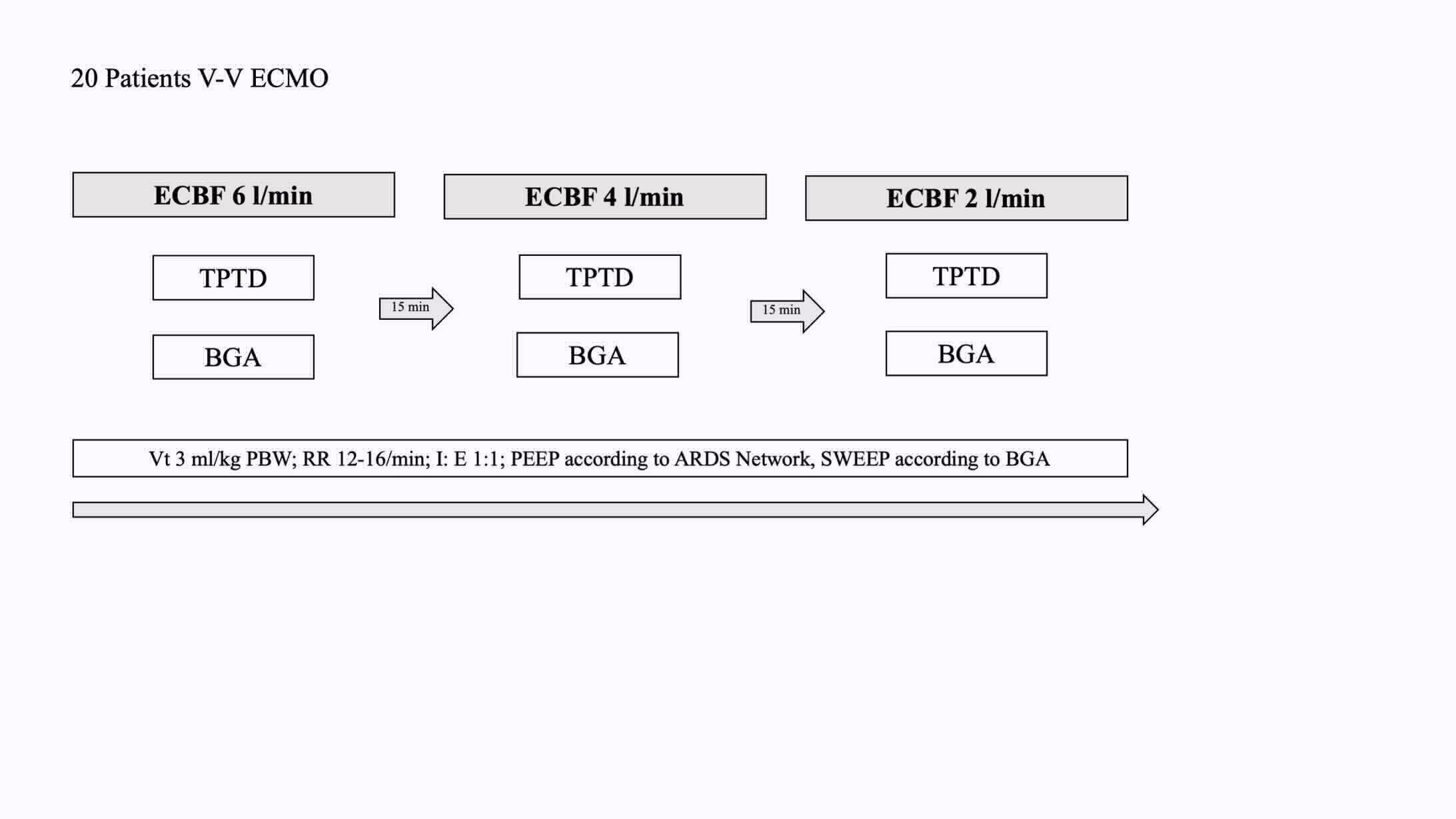


ECBF, *extracorporeal blood flow;* TPTD, *transpulmonary thermodilution*; BGA, *blood gas analysis*; Vt, *tidal volume*; PBW, *predicted body weight*; RR, *respiratory rate*, PEEP, *positive end-expiratory pressure*; ARDS*, acute respiratory distress syndrome*; SWEEP, *gas flow on the extracorporeal membrane*

**SI table 1:** Physiological data of 20 patients with severe ARDS managed with V-V ECMO at three different extracorporeal blood flows

|  | **ECBF**  **6 l/min** | **ECBF**  **4 l/min** | **ECBF**  **2 l/min** | **ECBF effect** |
| --- | --- | --- | --- | --- |
|  |  |  |  |  |
| **V_t_ [ml]** | 242 ± 79 | 242 ± 79 | 242 ± 79 | p = 1.000 |
| **Ppeak [cm H_2_O]** | 22 ± 4 | 22 ± 3 | 22 ± 3 | p = 0.754 |
| **PEEP [cm H_2_O]** | 13 ± 3 | 13 ± 3 | 13 ± 3 | p = 1.000 |
| **Respiratory rate (1/min)** | 12 ± 1 | 12 ± 1 | 12 ± 1 | p = 1.000 |
| **HR [1/min]** | 79 ± 17 **^b^** | 82 ± 19 **^c^** | 85 ± 20 | **p < 0.001** |
| **SV [ml]** | 85 ± 39 **^a,b^** | 86 ± 31 **^c^** | 96 ± 31 | **p < 0.001** |
| **CO_TPTD_ [l/min]** | 6.4 ± 2.3 **^b^** | 6.5 ± 2.2 **^c^** | 7.7 ± 2.2 | **p < 0.001** |
| **MAP [mmHg]** | 81 ± 11 **^a,b^** | 76 ± 10 | 77 ± 10 | **p = 0.004** |
| **CVP [mmHg]** | 15 ± 8 | 15 ± 5 | 16 ± 6 | p = 0.261 |
| **pHa** | 7.4 ± 0.1 **^a,b^** | 7.3 ± 0.1 **^c^** | 7.3 ± 0.1 | **p < 0.001** |
| **PaO_2_ [mmHg]** | 193 ± 94 **^a,b^** | 161 ± 78 **^c^** | 107 ± 68 | **p < 0.001** |
| **PaCO_2_ [mmHg]** | 52.9 ± 7.0 **^a,b^** | 55.4 ± 8.3 **^c^** | 63.4 ± 13.9 | **p < 0.001** |

V-V ECMO, *veno-venous extracorporeal membrane oxygenation*; ECBF, *extracorporeal blood flow*; V_t_, *tidal volume*; Ppeak, *peak airway pressure*; PEEP, *positive end-expiratory pressure*; HR, *heart rate*; SV, *cardiac stroke volume*; CO**_TPTD_**, *cardiac output measured by transpulmonary thermodilution*; MAP, *mean arterial pressure*; CVP, c*entral venous pressure*; pHa, *arterial pH*; PaO_2_, *arterial partial pressure of oxygen*; PaCO_2_, *arterial* *partial pressure of carbon dioxide*

Physiological data of 20 patients with severe ARDS managed with V-V ECMO at three ECBF. Values are means ± standard deviation. Repeated measures ANOVA followed by Holm-Sidak’s post-hoc test respectively Friedman procedure as appropriate was used to compare three different ECBF (*p* < 0.05). Bold numbers represent statistically significant differences between ECBF.

**a:** ECBF of **6** l/min vs. ECBF of **4** l/min

**b:** ECBF of **6** l/min vs. ECBF of **2** l/min

**c:** ECBF of **4** l/min vs. ECBF of **2** l/min

**SI table 2:** Calculated intrathoracic blood volume and extra-vascular lung water of 20 patients with severe ARDS managed with V-V ECMO at three different ECBF

|  | **ECBF**  **6 l/min** | **ECBF**  **4 l/min** | **ECBF**  **2 l/min** | **ECBF effect** |
| --- | --- | --- | --- | --- |
| **ITBV [ml]** | 1657 (1275-2404) **^a, b^** | 1716 (1299-2156) **^c^** | 1936 (1496-2553) | **p < 0.001** |
| **EVLW [ml]** | 2324 (2138-2788) **^a, b^** | 2370 (1563-2724) **^c^** | 1296 (1035-1631) | **p < 0.001** |

V-V ECMO, *veno-venous extracorporeal membrane oxygenation*; ECBF, *extracorporeal blood flow*; ITBV, *intrathoracic blood volume*; EVLW, *extravascular lung water*

Parameters calculated from transpulmonary thermodilution of 20 patients with severe ARDS managed with V-V ECMO at three different ECBF. Values are median (25% to 75% interquartile range). The Friedman procedure was used to compare three different ECBF (*p* < 0.05). Bold numbers represent statistically significant differences between ECBF.

**a:** ECBF of **6** l/min vs. ECBF of **4** l/min

**b:** ECBF of **6** l/min vs. ECBF of **2** l/min

**c:** ECBF of **4** l/min vs. ECBF of **2** l/min

**SI table 3:** Intra-examination characteristics of TPTD measurements of intrathoracic blood volume index and extra-vascular lung water index of 20 patients with severe ARDS managed with V-V ECMO at three different ECBF

|  | **ECBF**  **l/min** | **Coefficient of variation** | **Coefficient of error** | **Precision** | **Least significant change**  **%** |
| --- | --- | --- | --- | --- | --- |
| **ITBVI** | 6 | 0.04 (0.03 – 0.07) | 0.02 (0.02 – 0.04) | 0.05 (0.03 – 0.07) | 7 (5 – 10) |
| **ITBVI** | 4 | 0.04 (0.02 – 0.08) | 0.02 (0.01 – 0.04) | 0.04 (0.02 – 0.09) | 6 (3 – 12) |
| **ITBVI** | 2 | 0.04 (0.01 – 0.05) | 0.02 (0.01 – 0.03) | 0.03 (0.01 – 0.06) | 5 (2 – 9) |
| **EVLWI** | 6 | 0.03 (0.02 – 0.05) | 0.01 (0.01 -0.03) | 0.03 (0.02 – 0.05) | 4 (3 – 7) |
| **EVLWI** | 4 | 0.02 (0.01 – 0.04) | 0.01 (0.01 – 0.02) | 0.02 (0.01 – 0.04) | 3 (2 – 6) |
| **EVLWI** | 2 | 0.03 (0.01 – 0.04) | 0.02 (0.02 – 0.02) | 0.04 (0.04 – 0.04) | 4 (2 – 6) |

TPTD, *transpulmonary thermodilution*; V-V ECMO, *veno-venous extracorporeal membrane oxygenation*; ECBF, *extracorporeal blood flow*; ITBVI, *intrathoracic blood volume index*; EVLWI, *extravascular lung water index*

Parameters calculated from transpulmonary thermodilution of 20 patients with severe ARDS managed with V-V ECMO at three different ECBF. Values are median (25% to 75% interquartile range).

**Institutional management strategy for patients on V-V ECMO support**

Indications for V-V ECMO

In agreement with the guidelines established by the Extracorporeal Life Support Organization (ELSO) [1] and the ECMO to rescue Lung Injury in severe ARDS (EOLIA) trial [2], V-V ECMO is initiated in fully sedated patients (Richmond Agitation-Sedation Scale -5) with

- arterial partial pressure of oxygen / fraction of inspired oxygen (PaO_2_/FiO_2_) less than 50 mmHg for longer than 3 hours or
- PaO_2_/FiO_2_ less than 80 mmHg for longer than 6 hours or persistent acidosis (arterial pH less than 7.25 and arterial partial pressure of carbon dioxide (PaCO_2_) greater than 60 mmHg for longer than 6 hours)

despite protective mechanical ventilation (a tidal volume of 6 ml/kg, a positive end expiratory pressure (PEEP) adjustment according to the lowest elastance of the respiratory system and a driving pressure less than 15 cm H_2_O) and prone positioning [2-6]. Prone positioning and neuromuscular blocking agents are prescribed according to the attending physician.

Furthermore, V-V ECMO is considered in severe acute respiratory distress syndrome (ARDS) persistence without clinical improvement despite protective mechanical ventilation and at least two cycles of prone positioning [2, 7]. We will not initiate V-V ECMO support in patients who prefer palliative support, in patients with known end-stage chronic cardiopulmonary failure and in patients with an expected survival of less than 24 hours determined by the attending physician.

Cannulation strategy

The standard cannulation uses a 29 French multi-stage drainage cannula (HLS Cannula, Maquet, Rastatt, Germany) and a 23 French venous return cannula (HLS Cannula, Maquet, Rastatt, Germany), which are inserted through the right femoral and jugular veins, respectively. The V-V ECMO circuit is completed with a magnetically levitated rotor pump (Centrimag Circulatory Support System, Abbot, GmbH, Wiesbaden, Germany) and gas exchange membrane (PLS System, Maquet, Rastatt, Germany).

ECMO management

The V-V ECMO blood and gas flow are adjusted to obtain an arterial PaO_2_ between 65-90 mmHg and an arterial pH of 7.35 – 7.45 [2].

During ECMO support, the ventilator is set to a volume-controlled mode with a tidal volume of 2 ml per kilogram of ideal bodyweight, a respiratory rate of 12 per minute and a fraction of inspired oxygen of 40%. PEEP is titrated according to the lowest elastance of the respiratory system as described previously [8].

If arterial lactate level start to increase (greater than 2 mmol/L) under V-V ECMO support, a passive leg raised test in conjunction with echocardiographic assessment is performed to evaluate cardiac preload and fluid responsiveness. A lack of fluid responsiveness requires further echocardiographic assessment to rule out right or left ventricular failure.

A positive fluid balance in patients with ARDS has been associated with an increased mortality and duration of mechanical ventilation [9-11]. Thus, after an initial V-V ECMO stabilization period, we use diuretics or hemodialysis to facilitate a negative fluid balance. Afterward, we promote a spontaneous breathing by tapering the analgosedation. If clinically feasible, a V-V ECMO weaning trial is performed by reducing the V-V ECMO gas flow to 0 l/min for at least 24 hours. V-V ECMO support is discontinued if the PaO_2_ is higher than 70 mmHg and the arterial pH is greater than 7.25 with fraction of inspired oxygen less than 60% and an inspiratory plateau pressure less than 30 cm H_2_O have been achieved [2, 8].

**Calculations**

ITBV and ITBVI are calculated by the TPTD device as follows [12].:

$ITBV =GEDV x 1.25= COTPTD x MTt-COTPTD x DSt\times1.25$

*ITBVI =* $\frac{ITBV}{body surface area}$

EVLW and EVLWI calculated by the TPTD device as follows [12].:

$$EVLW =ITTV-ITBV= - 0.25 x COTPTD x MTt+ 1.25 x COTPTD x DSt$$

*EVLWI =* $\frac{EVLW}{predicted bodyweight}$

**Abbreviations**

ARDS acute respiratory distress syndrome

BGA blood gas analysis

CO_TPTD_ cardiac output measured by transpulmonary thermodilution

CI cardiac index

CVP central venous pressure

DSt downslope time

ECBF extracorporeal blood flow

ELSO Extracorporeal Life Support Organization

EVLW extravascular lung water

EVLWI extravascular lung water index

GEDV global end-diastolic volume

HR heart rate

ITBV intrathoracic blood volume

ITBVI intrathoracic blood volume index

ITTV intrathoracic thermovolume

MAP mean arterial pressure

MTt mean transit time

PBW predicted body weight

PEEP positive end-expiratory pressure

P_peak_ peak airway pressure

pHa arterial pH

PaCO_2_ arterial partial pressure of carbon dioxide

PaO_2_ arterial partial pressure of oxygen

PTV pulmonary thermovolume

RESP Respiratory ECMO Survival Prediction

RR respiratory rate

SAPS II Simplified Acute Physiology Score II

SI Supplementary Information

SOFA Sequential Organ Failure Assessment

SV stroke volume

SWEEP gas flow on the extracorporeal membrane

TPTD transpulmonary thermodilution

V_T_ tidal volume

V-V ECMO veno-venous extracorporeal membrane oxygenation

**References:**

1. Tonna J E, Abrams D, Brodie D, Greenwood J C, Rubio Mateo-Sidron J A, Usman A, Fan E (2021): Management of Adult Patients Supported with Venovenous Extracorporeal Membrane Oxygenation (VV ECMO): Guideline from the Extracorporeal Life Support Organization (ELSO). Asaio j 67, 6, 601-610. <http://dx.doi.org/10.1097/mat.0000000000001432>.

2. Combes A, Hajage D, Capellier G, Demoule A, Lavoue S, Guervilly C, Da Silva D, Zafrani L, Tirot P, Veber B, Maury E, Levy B, Cohen Y, Richard C, Kalfon P, Bouadma L, Mehdaoui H, Beduneau G, Lebreton G, Brochard L, Ferguson N D, Fan E, Slutsky A S, Brodie D, Mercat A, Eolia Trial Group R, Ecmonet (2018): Extracorporeal Membrane Oxygenation for Severe Acute Respiratory Distress Syndrome. N Engl J Med 378, 21, 1965-1975. <http://dx.doi.org/10.1056/NEJMoa1800385>.

3. Amato M B, Meade M O, Slutsky A S, Brochard L, Costa E L, Schoenfeld D A, Stewart T E, Briel M, Talmor D, Mercat A, Richard J C, Carvalho C R, Brower R G (2015): Driving pressure and survival in the acute respiratory distress syndrome. N Engl J Med 372, 8, 747-55. <http://dx.doi.org/10.1056/NEJMsa1410639>.

4. Shekar K, Badulak J, Peek G, Boeken U, Dalton H J, Arora L, Zakhary B, Ramanathan K, Starr J, Akkanti B, Antonini M V, Ogino M T, Raman L, Barret N, Brodie D, Combes A, Lorusso R, MacLaren G, Muller T, Paden M, Pellegrino V, Group E G W (2020): Extracorporeal Life Support Organization Coronavirus Disease 2019 Interim Guidelines: A Consensus Document from an International Group of Interdisciplinary Extracorporeal Membrane Oxygenation Providers. ASAIO J 66, 7, 707-721. <http://dx.doi.org/10.1097/MAT.0000000000001193>.

5. Brower R G, Lanken P N, MacIntyre N, Matthay M A, Morris A, Ancukiewicz M, Schoenfeld D, Thompson B T, National Heart L, Blood Institute A C T N (2004): Higher versus lower positive end-expiratory pressures in patients with the acute respiratory distress syndrome. N Engl J Med 351, 4, 327-36. <http://dx.doi.org/10.1056/NEJMoa032193>.

6. Krebs J, Pelosi P, Rocco P R M, Hagmann M, Luecke T (2018): Positive end-expiratory pressure titrated according to respiratory system mechanics or to ARDSNetwork table did not guarantee positive end-expiratory transpulmonary pressure in acute respiratory distress syndrome. J Crit Care 48, 433-442. <http://dx.doi.org/10.1016/j.jcrc.2018.10.005>.

7. Schmidt M, Pham T, Arcadipane A, Agerstrand C, Ohshimo S, Pellegrino V, Vuylsteke A, Guervilly C, McGuinness S, Pierard S, Breeding J, Stewart C, Ching S S W, Camuso J M, Stephens R S, King B, Herr D, Schultz M J, Neuville M, Zogheib E, Mira J P, Roze H, Pierrot M, Tobin A, Hodgson C, Chevret S, Brodie D, Combes A (2019): Mechanical Ventilation Management during Extracorporeal Membrane Oxygenation for Acute Respiratory Distress Syndrome. An International Multicenter Prospective Cohort. Am J Respir Crit Care Med 200, 8, 1002-1012. <http://dx.doi.org/10.1164/rccm.201806-1094OC>.

8. Graf P T, Boesing C, Brumm I, Biehler J, Muller K W, Thiel M, Pelosi P, Rocco P R M, Luecke T, Krebs J (2022): Ultraprotective versus apneic ventilation in acute respiratory distress syndrome patients with extracorporeal membrane oxygenation: a physiological study. J Intensive Care 10, 1, 12. <http://dx.doi.org/10.1186/s40560-022-00604-9>.

9. Shah A, Menaker J, Mazzeffi M A, Galvagno S M, Jr., Deatrick K B, Madathil R J, Rector R, O'Connor J V, Scalea T M, Tabatabai A (2021): Association of Volume Status During Veno-Venous Extracorporeal Membrane Oxygenation with Outcome. ASAIO J. <http://dx.doi.org/10.1097/MAT.0000000000001642>.

10. National Heart L, Blood Institute Acute Respiratory Distress Syndrome Clinical Trials N, Wiedemann H P, Wheeler A P, Bernard G R, Thompson B T, Hayden D, deBoisblanc B, Connors A F, Jr., Hite R D, Harabin A L (2006): Comparison of two fluid-management strategies in acute lung injury. N Engl J Med 354, 24, 2564-75. <http://dx.doi.org/10.1056/NEJMoa062200>.

11. Seitz K P, Caldwell E S, Hough C L (2020): Fluid management in ARDS: an evaluation of current practice and the association between early diuretic use and hospital mortality. J Intensive Care 8, 78. <http://dx.doi.org/10.1186/s40560-020-00496-7>.

12. SE P M S PiCCO Technologie Hämodynamisches Monitoring. <https://www.getinge.com/dam/hospital/documents/marketing-sales/brochures/german/picco_haemodynamisches_monitoring_broschuere-de-non_us.pdf>.
